# The Protective Effect of Dietary Phytosterols on Cancer Risk: A Systematic Meta-Analysis

**DOI:** 10.1155/2019/7479518

**Published:** 2019-06-23

**Authors:** Lu Jiang, Xin Zhao, Jun Xu, Chujun Li, Yue Yu, Wei Wang, Lingjun Zhu

**Affiliations:** ^1^Department of Oncology, The First Affiliated Hospital of Nanjing Medical University, Nanjing, China; ^2^Department of Respiratory and Critical Care Medicine, The First Affiliated Hospital of Nanjing Medical University, Nanjing, China; ^3^Department of Plastic and Burns Surgery, The First Affiliated Hospital of Nanjing Medical University, Nanjing, China; ^4^Department of Cardiovascular Medicine, The First Affiliated Hospital of Nanjing Medical University, Nanjing, China; ^5^Department of Thoracic Surgery, The First Affiliated Hospital of Nanjing Medical University, Nanjing, China; ^6^Department of Oncology, Sir Run Run Hospital, Nanjing Medical University, Nanjing, China

## Abstract

**Backgrounds/Aims:**

Many studies have explored the association between dietary phytosterols and cancer risk, but the results have been inconsistent. We aimed to provide a synopsis of the current understanding of phytosterol intake for cancer risk through a systematic evaluation of the results from previous studies.

**Methods:**

We performed a literature search of PUBMED, EMBASE, CNKI, and Wanfang, and studies published before May 2019 focusing on dietary total phytosterols, *β*-sitosterol, campesterol, stigmasterol, *β*-sitostanol, and campestanol, as well as their relationships with cancer risk, were included in this meta-analysis. Summaries of the relative risks from 11 case-control and case-cohort studies were eventually estimated by randomized or fixed effects models.

**Results:**

The summary relative risk for the highest versus the lowest intake was 0.63 (95% confidence interval [CI] = 0.49–0.81) for total phytosterols, 0.74 (95% CI = 0.54–1.02) for *β*-sitosterol, 0.72 (95% CI = 0.51–1.00) for campesterol, 0.83 (95% CI = 0.60–1.16) for stigmasterol, 1.12 (95% CI = 0.96–1.32) for *β*-sitostanol, and 0.77 (95% CI = 0.65–0.90) for campestanol. In a dose-response analysis, the results suggested a linear association for campesterol and a nonlinear association for total phytosterol intake.

**Conclusion:**

Our findings support the hypothesis that high phytosterol intake is inversely related to risk of cancer. Further studies with prospective designs that control for vital confounders and investigate the important anticancer effects of dietary phytosterols are warranted.

## 1. Introduction

Based on GLOBOCAN estimates, there were 14.1 million new cancer cases and 8.2 million cancer deaths worldwide in 2012 [1]. Cancer is a serious global health problem and has become one of the primary causes of death. The increasing trend in cancer globally could be slowed and reversed if preventive measures could provide a feasible approach [2].

The consumption of fruits and vegetables is considered to be inversely related to the risk of developing many chronic diseases. As we know, vegetables and fruits contain antioxidant phytochemicals that are thought to contribute to these health benefits [3–7]. Phytosterols are specific phytochemicals only found in plants that are structurally similar to cholesterol, except for an additional hydrocarbon chain at the C-24 position [8–10]. The number of foods that contain phytosterols is quite limited, but this is to be expected [11]. Campesterol, *β*-sitosterol, and stigmasterol are the three most common sterols, while *β*-sitostanol and campestanol are the two most common stanols [12]. It has been reported that phytosterols have protective effects on various chronic ailments including cardiovascular diseases [13–15] and diabetes [16]. Moreover, it is suggested that diets rich in phytosterols can reduce the risk of cancer by 20% [17–19]. Phytosterols and their oxy-derivatives may offer protection to the human body and inhibit cell proliferation and metastasis [20, 21], as well as the induction of apoptosis [22, 23], all of which have been experimentally verified. In addition, phytosterols may also be important in host systems and exert antitumor effects by improving the immune system's identification of cancer, affecting hormone-dependent (hormone-dependent) endocrine tumor growth, and regulating sterol biosynthesis [24–27].

A large number of dose-response meta-analyses have been performed to confirm the relationship of phytochemical consumption, including vitamin C [28–30], folate [31–33], and *β*-carotene [34–36], and cancer risk. However, although it has been hypothesized that high intakes of various phytochemicals could reduce the risk of cancer, no meta-analysis concerning the protective effects of total phytosterols, including *β*-sitosterol, campesterol, stigmasterol, *β*-sitostanol, and campestanol, on tumors has been performed. Therefore, it is of great importance to obtain evidence of the association between dietary phytosterols and cancer risk. The aim of our study was to assess the evidence from literature on the relationship between dietary phytosterol intake and cancer risk.

## 2. Discussion

In our meta-analysis, the highest versus the lowest intake of total phytosterols and campesterol were significantly and statistically associated with a reduction in cancer risk. After integrating the available evidence, a linear inverse association regarding dietary consumption and the risk of cancer was detected only for campesterol. Moreover, we found the first global nonlinear association between total phytosterol intake and cancer risk.

The mechanisms by which phytosterol consumption enables anticancer responses are varied and not fully understood. In vitro, a large number of experimental animal studies and human nutrition studies have been conducted to provide biological plausibility for the hypothesis. The inhibitory effect of *β*-sitosterol on tumor growth has been shown in various human tumor cell lines, including human colon cancer cell line HT116 [22], human lung cancer cell line A549 [37], human hepatic cancer cell line HepG2 [38], human prostate cancer cell lines PC-3 [39] and LNCaP [40], and human breast cancer cell lines MDA-MB-231 and MCF-7 [41, 42]. Furthermore, considerable interest has been developed through several animal studies dealing with the role of phytosterols in cancer protection. Ramalingam et al. [43] observed that the oral administration of *β*-sitosterol (20 mg/kg, three times per week for 24 weeks) was associated with the inhibition of proliferation and metastasis and the induction of apoptosis in renal cancer cells in rats. Similar results have also been observed in other animal experiments, which confirmed the antitumor effects of high intakes of phytosterols [44–48]. It has been shown that the consumption of phytosterols provides potential anticancer properties via various mechanisms, including the downregulation of cholesterol synthesis [49], the inhibition of cell cycle progression, cell invasion, migration, and adhesion [19, 50], the promotion of cell apoptosis [51], and the stimulation of the immune function. The inhibition of tumor growth by *β*-sitosterol can be explained by two pathways involving protein kinase C and the sphingomyelin cycle [52, 53]. Initial observations show that dietary phytosterols play a role of immunomodulatory compounds, in which mixtures of sterols and sterolins enhance the cell responsiveness of T-lymphocytes both in vitro and in vivo [25]. In addition, phytosterols, which are lipid components of membranes, are thought to influence membrane fluidity, levels of sex hormones [54], and NF-KB activation [26], all of which may play vital roles in cancer risk.

We observed a significant reduction in the risk of digestive system tumors via total phytosterol consumption, but not in the risk of reproductive system tumors. Stratified analysis indicated that the results were inconsistent with the pooled results of studies adjusted for body mass index (BMI), weight, or waist-to-hip ratio, which can be explained by a large number of studies that have confirmed the possible effect of weight on cancer risk [3, 14, 55]. The positive relationship between phytosterol consumption and cancer risk obtained in this meta-analysis of observational studies varied depending on geographic location. The pooled relative risk (RR) of cancer risk was 0.63 (95% confidence interval [CI] = 0.44–0.90) in subgroup America and 1.11 (95% CI = 0.70–1.75) in subgroup Europe for campesterol consumption. Similarly, the pooled RR was 0.60 (95% CI = 0.38–0.95) in subgroup America and 1.18 (95% CI = 0.69–1.01) in subgroup Europe for stigmasterol consumption. These differences may be due to genetic diversity and various dietary habits. Moreover, we found that high phytosterol intake was not a significant protective factor against tumors except those in the digestive system, which is in accordance with the importance of phytosterol intake on cholesterol and bile acid metabolism [9]. However, a larger number of studies are required to further explore whether the antitumor effect of phytosterol intake is influenced by cancer type.

This study had the following strengths. (1) As far as we know, our study is the first to comprehensively and systematically assess the relationship between dietary phytosterol intake and cancer risk. (2) The search strategy applied in this meta-analysis was based on professional search guidance and the studies included were of high quality. (3) The exposed populations in the studies were representative, which may have helped to reduce the heterogeneity in the analysis. (4) One of the authors conducted an independent assessment of each cancer site report to determine the eligibility of each article for inclusion in the meta-analysis. (5) Most studies included in this meta-analysis were adjusted for multiple vital confounders such as alcohol intake, smoking, BMI, family history of cancer, and energy intake. Studies were ruled out if there was no adjustment for age. (6) All studies ascertained outcomes using histological findings, which was the most reliable diagnostic criteria for cancer. (7) The results of this analysis were reliable and robust after conducting a systematic and comprehensive sensitivity analysis. In addition, the considerable dose-response effect was explored between total phytosterol and campesterol consumption and cancer risk.

However, there were some possible limitations in this meta-analysis. First was the heterogeneity. To compensate for this shortcoming, potential sources of heterogeneity were examined and detected in stratified and metaregression analysis by geographic location, cancer type, sex, fractions, number of cases, and adjustment for several confounders. Second, uneven distribution of the highest and lowest intakes of phytosterols may have resulted in heterogeneity in the summary analysis and reduced the reliability of the conclusions. Third, almost all of the studies included in our meta-analysis used food frequency questionnaires (FFQ) or a validated FFQ to collect dietary information except one conducted by Walcott et al. [56], which used the National Cancer Institute's Health Habits and History Questionnaire (HHHQ). Fourth, validated data were not available for dietary questionnaires in several studies; however, we conducted a stratified analysis and found no significant change in the association between dietary phytosterol consumption and cancer risk. Fifth, it was possible that the observed inverse association concerning the relationship could proceed from residual or unmeasured confounders. Higher dietary phytosterol consumption may relate to other lifestyle factors, including obesity and lower prevalence of smoking, lower consumption of alcohol, and other potential confounders. Most but not all related confounders were adjusted for in the studies. Sixth, there was evidence of a nonlinear association between dietary total phytosterol consumption and cancer risk but a linear association between dietary campesterol consumption and cancer risk. However, we could not suggest a dose-response effect for the consumption of dietary campestanol. This was due to the small number of studies and the data available in our analysis. There was no need to conduct a dose-response analysis between dietary *β*-sitosterol, stigmasterol, and *β*-sitostanol intake and cancer risk because our results showed that there were no inverse associations. Thus, further research is required to investigate whether there are linear or nonlinear associations between *β*-sitosterol, stigmasterol, *β*-sitostanol, and campestanol and risk of cancer. In addition, we only included observational studies in our meta-analysis and most were case-control studies that were prone to selective bias, recollection bias, and inaccuracies. Finally, almost all studies published to date have been conducted primarily among middle-aged and older persons; thus, we cannot exclude the possibility that phytosterol intake during an earlier period of life might has a stronger protective effect against cancer.

Cancer is one of the main causes of early death; however, the association between dietary levels of phytosterols and cancer development is still controversial. The results in our meta-analysis suggested that a 500 mg/day consumption of dietary total phytosterol minimized the risk of cancer and a 10 mg/day increment in dietary campesterol decreased the risk of tumorigenesis by 13%. Dietary sources of phytosterol are mainly seeds, cereals, legumes, vegetable oils, and nuts [55, 57]. In the United States, the daily dietary intakes of phytosterols are 160–360 mg/day [58], which means that a majority of people will not reduce their risk of cancer via phytosterol intake. However, a well-designed diet containing appropriate increases in the foods mentioned above may reduce the risk of cancer.

To conclude, our results suggest that there is a significant and nonlinear inverse association between dietary phytosterol consumption and the risk of cancer, with the greatest reduction in risk found when the intake has increased from a very low level. In addition, the first meta-analysis of the association between cancer risk and phytosterol intake to date in our study not only summarized the current literature on the epidemiology of diet and cancer risk, but also provided evidence to support a healthy diet. Large sample sizes and well-designed studies adjusted for important confounders are needed to further confirm our results.

## 3. Material and Methods

### 3.1. Search Strategy and Selection Criteria

**Figure 1:**
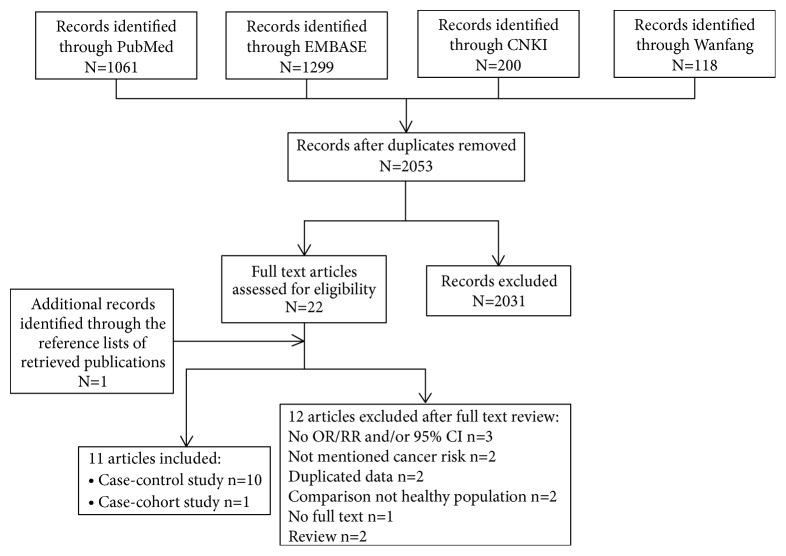
The process diagram of article search and selection in the meta-analysis.

A comprehensive, computerized literature search regarding the association between dietary phytosterol consumption and cancer risk was conducted in four databases (PubMed, EMBASE, Wanfang, and CNKI) and studies published before May 2019 were included in this analysis. The MeSH terms combined for the search were specifically as follows: (((((((((phytosterols[MeSH Terms]) OR phytosterols[Title/Abstract])) OR *β*-sitosterol[Title/Abstract]) OR campesterol[Title/Abstract]) OR stigmasterol[Title/Abstract]) OR *β*-sitostanol[Title/Abstract]) OR campestanol[Title/Abstract])) AND ((((((cancer[MeSH Terms]) OR cancer[Title/Abstract])) OR neoplasms[Title/Abstract]) OR carcinoma[Title/Abstract]) OR tumor[Title/Abstract]). It should be noted that duplicated results may be published in several articles. Hence, in such cases, we selected the most recent or most informative paper for our analysis. Moreover, we also scrutinized the references of retrieved publications to identify any potential missing studies. A flow chart of the search and selection of these studies is provided in Figure 1.

To be eligible for our analysis, the studies had to meet the following criteria: (1) published as an original study; (2) the study design was case-control or cohort; (3) the aim was to investigate the associations between the intake of total phytosterols or *β*-sitosterol, campesterol, stigmasterol, *β*-sitostanol or campestanol, and cancer risk; (4) the odds ratio (OR) or RR with the corresponding 95% CIs for the association between dietary total phytosterol or *β*-sitosterol, campesterol, stigmasterol, *β*-sitostanol or campestanol consumption, and cancer risk was reported (mechanistic research, animal experiments, and human feeding studies on cancers were excluded); (5) the RR or OR and the corresponding 95% CIs were at least adjusted for age.

### 3.2. Data Extraction and Quality Assessment

All data were independently extracted by two of the authors who ensured that the information complied with the selection criteria above. A standardized data collection protocol was used for data extraction: the name of the first author, publication year, cancer type, study design, geographic location, sex, number of cases and controls, median or mean age of participants, dietary assessment method (i.e., type, whether it was validated, and number of items), exposure and dietary intake levels, ORs or RRs, and the corresponding 95% CIs for the highest versus the lowest level of dietary intake and an adjustment for confounders in multivariate analysis.

A 9-star system according to the New-castle–Ottawa Scale [59] was used by two authors to assess study quality. Quality assessments were investigated based on the following features: selection, comparability, and exposure or outcome assessment. The maximum score of the three parameters was 4, 2, and 3 separately. The maximum total score was 9, with a score of 6 or lower indicating a low-quality study.

### 3.3. Statistical Analysis

To take within-study and between-study variations into consideration, both fixed effects models and random effects models were used to evaluate the pooled RRs and corresponding 95% CIs for the highest versus the lowest level of dietary phytosterol consumption and for the dose-response analysis. The analysis evaluated heterogeneity among the studies via the Cochran's Q test and* I*^*2*^ (inconsistency index) statistic [60]. Heterogeneity was considered significant if* P* < 0.10. A value less than 25% indicated a lack of heterogeneity among studies and the use of the fixed-effects model (the Mantel-Haenszel method) was allowed. If* I*^*2*^ value was greater than 50%, severe heterogeneity was considered, which indicated that the results could not be pooled together and discussed; thus a random effects model had to be used [61, 62]. Although both models drew similar conclusions, the true potential impacts on the results of the random effects model presented here were different [63].

We also evaluated whether potential publication bias existed in our analysis by using the Begg test [64] and the Egger's regression test [65]. Subgroup analysis based on geographic location, cancer type, sex, fractions, number of cases, and adjustment for confounders, such as alcohol, smoking, BMI, family history, and energy intake, were conducted in this meta-analysis to explore possible heterogeneity and to analyze whether there was a correlation among some subgroups [62]. We also performed a sensitivity analysis to estimate the potential effect of each individual study and the stability of the results by the leave-one-out method, which means consecutively eliminating each study from the analysis one at a time. Moreover, the metaregression analysis was examined to investigate the variables of several factors to explore the potential heterogeneity.

The methods developed by Orsini [66] and Greenland and Longneckre [67] were used for the dose-response analysis and linear trends, and the 95% CIs from the natural logs of the RRs and CIs across categories of dietary campesterol consumption were estimated. This means that studies providing data of the distributions of person-years or cases and noncases and the RR or OR with the variance estimates for at least 3 quantitative exposure categories of campesterol were required. It was acceptable if the mean or median values of campesterol intake in each category were reported using the variance-weighted least squares regression [66, 67]. If the mean or median values of categories of intake level were not available, we estimated the midpoint of each category as the average of the lower and upper boundaries. When the highest open-ended category was reported, we considered the width of the open interval as the same as that of the adjacent interval. If studies only provided the open-ended lowest category, the lowest boundary was assumed to be zero. A 10 mg/day increment for cancer risk reduction was revealed in the dose-response results.

We examined the relationship between total dietary phytosterol consumption and cancer risk using best-fit, second-order fractional polynomial models [68], defined as those with the least deviance for a potential nonlinear dose-response meta-analysis. We used a likelihood ratio test to determine the difference between linear and nonlinear models to further assess the nonlinearity [69].

STATA software (version 13.1; StataCorp, College Station, TX) was used for all statistical analyses involved. All* P* values were two sided and* P* < 0.05 was considered statistically significant.

## 4. Results

### 4.1. Search Results and Characteristics of the Studies

As shown in Figure 1, 11 studies [56, 70–79] with 15 comparisons were included according to the inclusion criteria, 10 studies were case-control study and 1 was case-cohort study. In aggregate analysis, the number of participants included in the 11 selected studies was 16,763, ranging from 295 to 3,615, including 9,134 American participants and 4,014 European participants; only 1 study enrolled participants in Asia (n = 3,615). The role that dietary phytosterol intake may play in cancer development was explored in lung, breast, endometrial, esophageal, gastric, colorectal, testicular, ovarian, and prostate cancers in these studies. In addition, six studies included men and women, two included only men, and two included only women. One study conducted by Normén et al. [75] investigated the influence of phytosterol intake on colon and rectal cancer, in males and females, respectively. The FFQ, validated only in 5 studies, was applied in all studies except one, which used the National Cancer Institute's HHHQ to assess diet. The intake levels of phytosterols were divided into at least three parts. Supplementary Table S1, which contains the dominant characteristics in the relevant literature, additionally shows a summary of quality scores of case-control studies, ranging from 7 to 9 out of a total of 10.

### 4.2. Dietary Total Phytosterols and Cancer Risk

**Figure 2:**
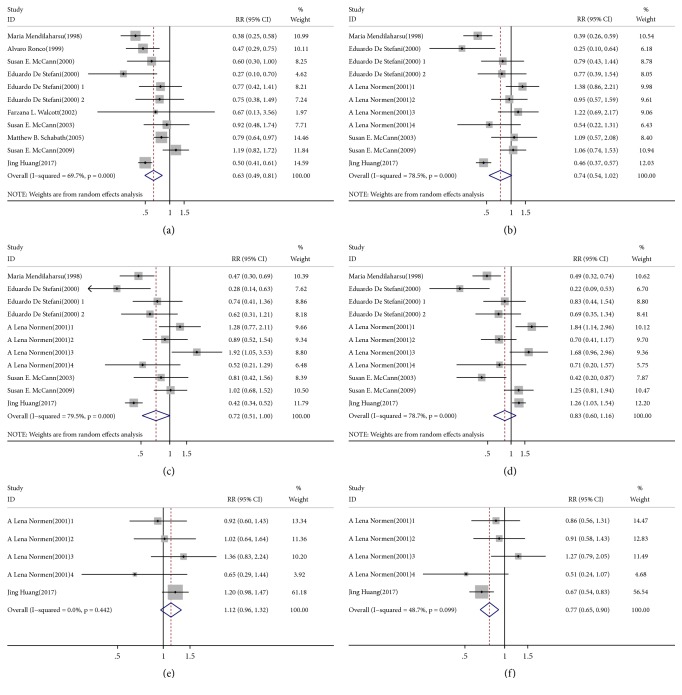
(a)* Forest plot of highest versus lowest categories of total phytosterol consumption on cancer risk*. RR, relative risk; CI, confidence interval; (b)* forest plot of highest versus lowest categories of β-sitosterol consumption on cancer risk.* RR, relative risk; CI, confidence interval; (c)* forest plot of highest versus lowest categories of campesterol consumption on cancer risk.* RR, relative risk; CI, confidence interval; (d)* forest plot of highest versus lowest categories of stigmasterol consumption on cancer risk.* RR, relative risk; CI, confidence interval; (e)* forest plot of highest versus lowest categories of β-sitostanol consumption on cancer risk.* RR, relative risk; CI, confidence interval; (f)* forest plot of highest versus lowest categories of campestanol consumption on cancer risk.* RR, relative risk; CI, confidence interval.

**Figure 3:**
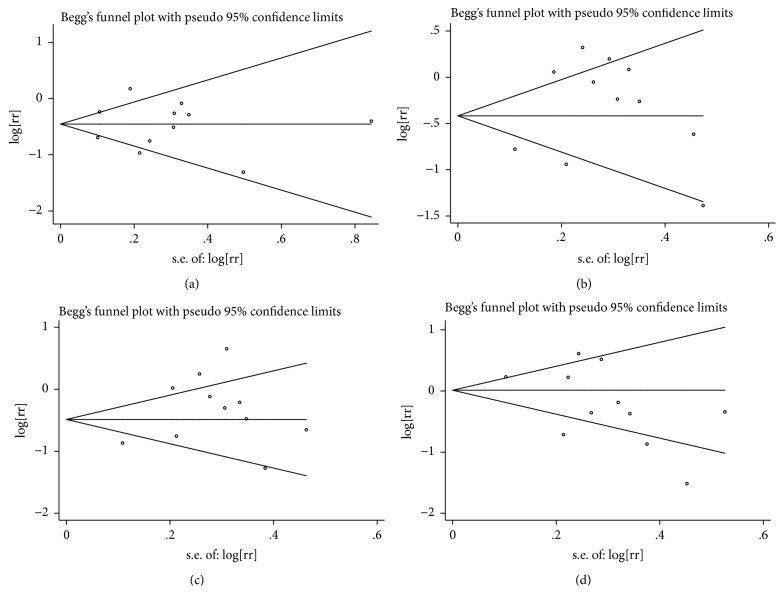
(a) Begg's funnel plot for publication bias test of the relationship between total phytosterol consumption and cancer risk. (b) Begg's funnel plot for publication bias test of the relationship between *β*-sitosterol consumption and cancer risk. (c) Begg's funnel plot for publication bias test of the relationship between campesterol consumption and cancer risk. (d) Begg's funnel plot for publication bias test of the relationship between stigmasterol consumption and cancer risk.

**Figure 4:**
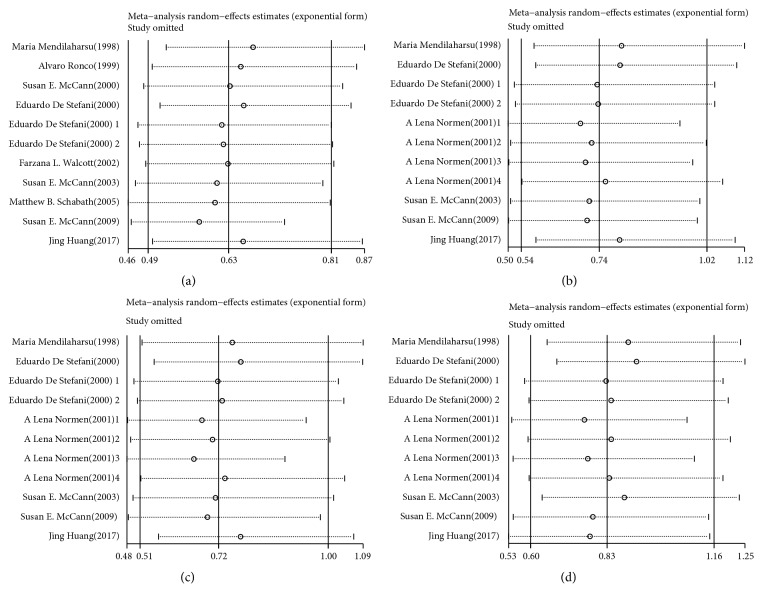
(a) Sensitivity analysis of highest versus lowest categories of total phytosterol consumption on cancer risk. (b) Sensitivity analysis of highest versus lowest categories of *β*-sitosterol consumption on cancer risk. (c) Sensitivity analysis of highest versus lowest categories of campesterol consumption on cancer risk. (d) Sensitivity analysis of highest versus lowest categories of stigmasterol consumption on cancer risk.

**Figure 5:**
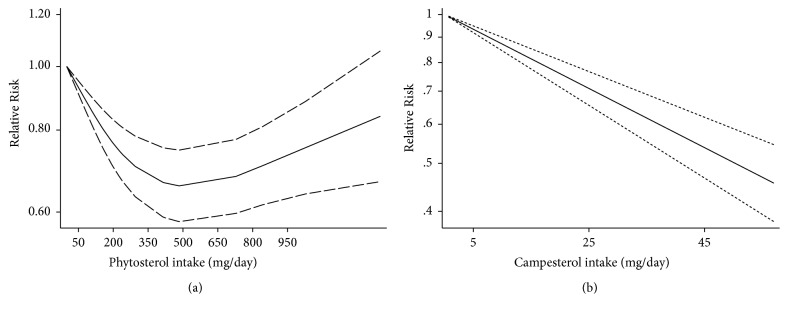
(a) Nonlinear dose-response analysis of the relationship between total phytosterol consumption and cancer risk. (b) Linear dose-response analysis of the relationship between campesterol consumption and cancer risk.

The inverse association between dietary total phytosterols and cancer risk based on 10 articles with 11 comparisons is shown in Figure 2(a). Severe heterogeneity was present among the 11 comparisons (*P* < 0.001,* I*^*2*^ = 69.7%), in which the overall pooled RR was 0.63 (95% CI = 0.49–0.81) for the highest versus the lowest total phytosterol intakes calculated according to the randomized effect model. No publication bias existed in our study based on the Begg's funnel plot (Figure 3(a)) and Egger's test (*P* = 0.936). Furthermore, the pooled result did not alter substantially after we omitted the studies one by one according to sensitivity analysis, which confirmed the stability of the inverse association (Figure 4(a)). On the other hand, metaregression analysis revealed that both sex and adjustment for family history (*P* = 0.022) were significant factors in the relationship between dietary total phytosterol consumption and tumorigenesis; sex and adjustment for family history combined could explain 100% of the estimated between-study variance (**τ**^***2***^**)**, which decreased from 0.0992 to 0. Thus, the two factors were considered as the primary sources of heterogeneity across the comparisons. We included 7 articles in our dose-response analysis. The evidence of a nonlinear association was observed, indicating that 500 mg/day dietary total phytosterol consumption could reduce the risk of cancer development; the details are shown in Figure 5.

The point estimate for the pooled RR in the majority of the subgroup analyses was < 1, except for the subgroup of the non-BMI adjustment (RR = 0.73; 95% CI = 0.47–1.14) and nondigestive system tumors (RR = 0.92; 95% CI = 0.65–1.30 for reproductive system tumors and RR = 0.56; 95% CI = 0.27–1.15 for respiratory system tumors). To conclude, dietary total phytosterol consumption played a strong, protective role against the development of cancer.

### 4.3. Dietary *β*-Sitosterol Consumption and Cancer Risk

The association between dietary *β*-sitosterol intake and cancer risk is shown in Figure 2(b). No association was obtained in our analysis using the randomized effect model because the pooled RR was 0.74 (95% CI = 0.54–1.12) with significant heterogeneity (*P* < 0.001,* I*^*2*^ = 78.5%). Begg's funnel plot (Figure 3(b)) and Egger's test (*P* = 0.279) indicated that there was no publication bias. It was suggested that the outcome of this meta-analysis was statistically reliable according to the sensitivity analysis (Figure 4(b)). To search for the sources of heterogeneity, we conducted a meta-regression analysis and found sex was the main source of heterogeneity as* τ*^*2*^ decreased from 0.2118 to 0. Hence, the main sources of heterogeneity concerning the association between dietary intake of *β*-sitosterol and cancer risk were obtained even though the heterogeneity was significant. In a stratified analysis, we observed that the consumption of high levels of dietary *β*-sitosterol may have positive effects on antitumor mechanisms if our meta-analysis included only results adjusted for BMI.

### 4.4. Dietary Campesterol Consumption and Cancer Risk

Figure 2(c) shows the pooled RR estimates (RR = 0.72; 95% CI = 0.51–1.00) of 7 articles with 11 comparisons for the highest versus the lowest campesterol intake based on the randomized effect model. Significant heterogeneity (*P* < 0.001,* I*^*2*^ = 79.5%) was observed in our study. According to Begg's funnel plot (Figure 3(c)) and Egger's test (*P* = 0.143), no publication bias was found. We also performed sensitivity analysis to find the main source of heterogeneity by leaving out each study subsequently one at a time. However, the heterogeneity was unchanged when any one study was excluded (Figure 4(c)). In addition, we carried out a metaregression analysis to find the main source of heterogeneity. It was shown that sex and adjustment for BMI were the main sources of significant heterogeneity and *τ*^*2*^ decreased from 0.2353 to 0.0331, which meant that the subgroups of sex and adjustment for BMI combined could explain 85.93% of the heterogeneity. Five studies were included in the dose-response analysis and a linear association was observed as each 10 mg/day increase of dietary campesterol consumption reduced cancer risk by 13% (RR = 0.87; 95% CI = 0.84–0.90).

Subgroup analysis was conducted to examine the sources of heterogeneity between the studies regarding the relationship between dietary campesterol intake and cancer risk. When stratifying by geographic location, no inverse association was revealed between campesterol intake and cancer risk in Europe (RR = 1.11; 95% CI = 0.70–1.75). When stratifying by sex and adjustment for confounders, there were no changes in the direction of the effect in a subgroup of males and females combined (RR = 0.47; 95% CI = 0.37–0.59) or an adjustment for BMI (RR = 0.55; 95% CI = 0.39–0.79). In summary, the cancer risk could be reduced by incremental increased levels of campesterol intake.

### 4.5. Dietary Stigmasterol Consumption and Cancer Risk

We identified 7 articles with 11 comparisons that were included in the analysis of the highest versus the lowest intake of stigmasterol and cancer risk using the randomized effect model. The pooled RR was 0.83 (95% CI = 0.49–0.81) with significant heterogeneity (*P *< 0.001,* I*^*2*^ = 78.7%), indicating that there was no relationship between dietary stigmasterol and cancer risk in our study. There was no publication bias based on Begg's funnel plot (Figure 3(d)) and Egger's test (*P* = 0.089). The sensitivity analysis conducted in this meta-analysis suggested that there were no changes in the direction of the effects of the results by the leave-one-out method, which confirmed the reliability and credibility of the results (Figure 4(d)). Additionally, there was evidence of a main source of significant heterogeneity in metaregression analysis. The subgroup of number of cases mainly accounted for heterogeneity because it could explain 38.4% of *τ*^*2*^, which decreased from 0.2262 to 0.1394. However, severe heterogeneity (*P* < 0.05,* I*^*2*^ = 64.7%) still existed in our analysis after metaregression analysis. In a stratified analysis, no inverse association was observed except for the geographic location, as the pooled RR was 0.60 (95% CI = 0.38–0.95) when the investigations were located in America.

### 4.6. Dietary *β*-Sitostanol Consumption and Cancer Risk

Two articles with five comparisons examined the association between dietary *β*-sitostanol intake and cancer development (2,693 cases among 7,629 participants). The pooled RR was 1.12 (95% CI = 0.96–1.32) for cancer risk with no evidence of heterogeneity (*P* = 0.442,* I*^*2*^ = 0).

### 4.7. Dietary Campestanol Consumption and Cancer Risk

Two articles with five comparisons were included in the analysis of the highest versus the lowest intake of campestanol and cancer risk. The pooled RR was 0.82 (95% CI = 0.63–1.06) for cancer risk with little evidence of heterogeneity (*P* = 0.099, *I*^2^ = 48.7%) based on 2,693 cases among 7,629 participants.

## Supplementary Materials

Supplementary Materials Supplementary Table S1. Characteristics of studies on dietary phytosterol consumption and cancer risk. Supplementary Table S2. Stratified analysis of dietary phytosterol consumption and cancer risk.
