# Supplementary material for: The Protective Effect of Dietary Phytosterols on Cancer Risk: A Systematic Meta-Analysis
Source: J Oncol. 2019 Jun 23;2019:7479518. doi: 10.1155/2019/7479518 (PMC6612402; doi:10.1155/2019/7479518)
Supplement: Supplementary Materials — Supplementary Table S1. Characteristics of studies on dietary phytosterol consumption and cancer risk. Supplementary Table S2. Stratified analysis of dietary phytosterol consumption and cancer risk. [file 7479518.f1.pdf]

First author, (year of publication)	Cancer type	Country; study design	Gender	Case&Control	age (y)	Dietary assessment	Exposure	Contrast (highest vs lowest)	Ajusted OR/RR(95%CI) (highest vs lowest)	Ajustment for confounders	quality scores
Maria Mendilaharsu(1998)	Lung cancer	Uruguay;case-control study	M&F	463&465	30–89 y	Validated FFQ;64 food items	Total phytosterols	Quartile highest vs lowest	0.38(0.25–0.58)	age, residence, urban/rural status, family history of lung cancer, tobacco smoking (pack-years), body mass index and total flavonoid intake	9
							β-Sitosterol	Quartile highest vs lowest	0.39(0.26–0.59)		
							Campesterol	Quartile highest vs lowest	0.47(0.30–0.69)		
							Stigmasterol	Quartile highest vs lowest	0.49(0.32–0.74)		
Alvaro Ronco(1999)	Breast Cancer	Uruguay;case-control study	M&F	400&405	20–89 y	FFQ;64 food items	Total phytosterols	Quartile highest vs lowest	0.47(0.29–0.75)	age, residence, urban/rural status, family history of breast cancer in a 1st-degree relative, body mass index, age at menarche, parity, menopausal status, total energy, and total vegetable intakes plus each nutrient	8
Susan E. McCann(2000)	endometrial cancer	USA;case-control study	F	232&639	40-85 y	FFQ;172 food items	Total phytosterols	Quartile ＞122 vs ＜67mg/d	0.60 (0.30–1.00)	age, education, BMI, diabetes, hypertension, pack-years cigarette smoking, age at menarche, parity, oral contraceptive use, menopause status, postmenopausal estrogen use and total energy	8
Eduardo De Stefani(2000)	Esophageal cancer	Uruguay;case-control study	M&F	111&444	40-89 y	Validated FFQ;64 food items	Total phytosterols	Quartile highest vs lowest	0.27 (0.10–0.70)	age, gender, residence, urban/rural status, education, body mass index, tobacco smoking, alcohol drinking, total energy intake and each antioxidant	8
							β-Sitosterol	Quartile highest vs lowest	0.25 (0.10–0.64)		
							Campesterol	Quartile highest vs lowest	0.28 (0.14–0.63)		
							Stigmasterol	Quartile highest vs lowest	0.22 (0.09–0.53)		
Eduardo De Stefani(2000) 1	Gastric cancer	Uruguay;case-control study	M&F	120&360	30-89 y	Validated FFQ;64 food items(for vegetables)	Total phytosterols	Tertile highest vs lowest	0.77 (0.42–1.41)	age,gender,residence,urban/ruralstatus,education,body mass index, total energy and each plant sterol	8
							β-Sitosterol	Tertile highest vs lowest	0.79 (0.43–1.44)		
							Campesterol	Tertile highest vs lowest	0.74 (0.41–1.36)		
							Stigmasterol	Tertile highest vs lowest	0.83 (0.44–1.54)		
Eduardo De Stefani(2000) 2	Gastric cancer	Uruguay;case-control study	M&F	120&360	30-89 y	Validated FFQ;64 food items(for fruit)	Total phytosterols	Tertile highest vs lowest	0.75 (0.38–1.49)	rage,gender,residence,urban/ruralstatus,education,body mass index, total energy and each plant sterol	8
							β-Sitosterol	Tertile highest vs lowest	0.77 (0.39–1.54)		
							Campesterol	Tertile highest vs lowest	0.62 (0.31–1.21)		
							Stigmasterol	Tertile highest vs lowest	0.69 (0.35–1.34)		
A Lena Normén(2001)1	Colon cancer	Netherlands;case-cohort Study	M	504&1525	55-69 y	FFQ;150 food items	β-Sitosterol	Quintile 286 vs 119mg/d	1.38 (0.86–2.21)	age, smoking (pack-years), alcohol use, family history of colorectal cancer, education level, cholecystectomy, and energy intake	9
							Campesterol	Quintile 81 vs 34mg/d	1.28 (0.77–2.11)		
							Stigmasterol	Quintile 39 vs 16mg/d	1.84 (1.14–2.96)		
							β-Sitostanol	Quintile 25 vs 8mg/d	0.92 (0.60–1.43)		
							Campestanol	Quintile 19 vs 5mg/d	0.86 (0.56–1.31)		

Continued

Supplementary Table S1. Characteristics of studies on dietary phytosterol consumption and cancer risk.

Supplementary Table S1

First author, (year of publication)	Cancer type	Country; study design	Gender	Case&Control	age (y)	Dietary assessment	Exposure	Contrast (highest vs lowest)	Ajusted OR/RR(95%CI) (highest vs lowest)	Ajustment for confounders	quality scores
A Lena Normén(2001)2	Colon cancer	Netherlands;case-cohort study	F	387&1598	55-69 y	FFQ;150 food items	β-Sitosterol	Quintile 242 vs 108mg/d	0.95 (0.57–1.59)	age, smoking (pack-years), alcohol use, family history of colorectal cancer, education level, cholecystectomy, and energy intake	9
							Campesterol	Quintile 68 vs 30mg/d	0.89 (0.52–1.54)		
							Stigmasterol	Quintile 32 vs 14mg/d	0.70 (0.41–1.17)		
							β-Sitostanol	Quintile 20 vs 7mg/d	1.02 (0.64–1.64)		
							Campestanol	Quintile 13 vs 5mg/d	0.91 (0.58–1.43)		
A Lena Normén(2001)3	Rectal cancer	Netherlands;case-cohort study	M	504&1525	55-69 y	FFQ;150 food items	β-Sitosterol	Quintile 286 vs 119mg/d	1.22 (0.69–2.17)	age, smoking (pack-years), alcohol use, family history of colorectal cancer, education level, cholecystectomy, and energy intake	9
							Campesterol	Quintile 81 vs 34mg/d	1.92 (1.05–3.53)		
							Stigmasterol	Quintile 39 vs 16mg/d	1.68 (0.96–2.96)		
							β-Sitostanol	Quintile 25 vs 8mg/d	1.36 (0.83–2.24)		
							Campestanol	Quintile 19 vs 5mg/d	1.27 (0.79–2.05)		
A Lena Normén(2001)4	Rectal cancer	Netherlands;case-cohort study	F	387&1598	55-69 y	FFQ;150 food items	β-Sitosterol	Quintile 242 vs 108mg/d	0.54 (0.22–1.31)	age, smoking (pack-years), alcohol use, family history of colorectal cancer, education level, cholecystectomy, and energy intake	9
							Campesterol	Quintile 68 vs 30mg/d	0.52 (0.21–1.29)		
							Stigmasterol	Quintile 32 vs 14mg/d	0.71 (0.20–1.57)		
							β-Sitostanol	Quintile 20 vs 7mg/d	0.65 (0.29–1.44)		
							Campestanol	Quintile 13 vs 5mg/d	0.51 (0.24–1.07)		
Farzana L. Walcott(2002)	Testicular cancer	USA;case-control study	M	159&136	18-55 y	Validated HHHQ;152 food items	Total phytosterols	Quartile ＞237.7 vs ＜ 71.8ug/1000kcal	0.67 (0.13–3.56)	ethnicity, age, education, income, history of cryptorchidism, early onset of puberty, history of severe adolescent acne, balding unrelated to cancer therapy, body mass index, total energy intake, total fat intake and total dietary fiber intake	8
Susan E. McCann(2003)	ovarian cancer	USA;case-control study	F	124&696	40-85 y	FFQ;unmentioned	Total phytosterols	Quintile ＞935 vs ＜ 179mg/d	0.92 (0.48–1.74)	age, education, total months menstruating, difficulty becoming pregnant, oral contraceptive use (ever/never), menopausal status, and total energy	7
							β-Sitosterol	Quintile ＞881 vs ＜ 135mg/d	1.09 (0.57–2.08)		
							Campesterol	Quintile ＞32 vs ＜ 15mg/d	0.81 (0.42–1.56)		
							Stigmasterol	Quintile ＞23 vs ＜ 12mg/d	0.42 (0.20–0.87)		
Matthew B. Schabath（2005）	Lung cancer	US;case-control study	M&F	1674&1735	62.1&61.5 y	Validated FFQ;unmentioned	Total phytosterols	Quartile ≤150.6 vs ≥ 664.7mg/d	0.79(0.64–0.97)	age, sex, ethnicity, smoking status, cigarettes smoked per day, years of smoking, education, income, body mass index, and total energy	9

Continued

Supplementary Table S1. Characteristics of studies on dietary phytosterol consumption and cancer risk.

Supplementary Table S1

First author, (year of publication)	Cancer type	Country; study design	Gender	Case&Control	age (y)	Dietary assessment	Exposure	Contrast (highest vs lowest)	Ajusted OR/RR(95%CI) (highest vs lowest)	Ajustment for confounders	quality scores
Susan E. McCann(2009)	Prostate cancer	USA;case-control study	M	433&538	NA	FFQ;172 food items	Total phytosterols	Quartile ＞562.2 vs ＜166.1mg/d	1.19 (0.82–1.72)	age, education, body mass index, cigarette smoking status, vegetable intake and total energy	9
							β-Sitosterol	Quartile ＞525.8 vs ＜126.8mg/d	1.06 (0.74–1.53)		
							Campesterol	Quartile ＞32.9 vs ＜17.4mg/d	1.02 (0.68–1.52)		
							Stigmasterol	Quartile ＞23.9 vs ＜13.8mg/d	1.25 (0.81–1.94)		
Jing Huang(2017)	Colorectal cancer	China;case-control study	M&F	1802&1813	30–75 y	Validated FFQ;81food items	Total phytosterols	Quartile 294·5 vs 154.8mg/d	0.50(0.41–0.61)	age, sex, marital status, residence, education, occupation, income level, BMI, smoking status, passive smoking, alcohol drinking, family history of cancer, occupational physical activity, household and leisure-time activities, dietary intake of dairy products, red and processed meat	9
							β-Sitosterol	Quartile 189.9 vs 97.4mg/d	0.46(0.37–0.57)		
							Campesterol	Quartile 57.0 vs 20.8mg/d	0.42(0.34–0.52)		
							Stigmasterol	Quartile 30.6 vs 16.5mg/d	1.26(1.03–1.54)		
							β-Sitostanol	Quartile 23.7 vs 12.5mg/d	1.20(0.98–1.47)		
							Campestanol	Quartile 4.6 vs 2.4mg/d	0.67(0.54–0.83)		

Supplementary Table S1. Characteristics of studies on dietary phytosterol consumption and cancer risk. FFQ, food frequency questionnaire; HHHQ, Health Habits and History Questionnaire.

Supplementary Table S2

Group	Total phytosterols					β-Sitosterol				
	No. of studies	Summary OR(95%CI)	χ^2	*P*	I^2(%)	No. of studies	Summary OR(95%CI)	χ^2	*P*	I^2(%)
Total	11	0.63(0.49–0.81)	33.00	0. 000	69.7	11	0.74(0.54–1.02)	46.57	0. 000	78.5
**Geographic location**										
Europe	0	-	-	-	-	4	1.07(0.78–1.48)	3.77	0.288	20.3
America	10	0.66(0.50–0.87)	24.66	0.003	63.5	6	0.67(0.43–1.05)	19.60	0.001	74.5
Asia	1	0.50(0.41–0.61)	0.00	-	-	1	0.46(0.37–0.57)	0.00	-	-
**Cancer type**										
Digestive system tumors	4	0.55(0.40–0.76)	4.61	0.203	34.9	8	0.74(0.49–1.09)	31.11	0.000	77.5
Reproductive system tumors	4	0.92(0.65–1.30)	3.83	0.280	21.7	2	1.07(0.78–1.47)	0.01	0.941	0.0
Respiratory system tumors	2	0.56(0.27–1.15)	9.34	0. 002	89.3	1	0.39(0.26–0.59)	0.00	-	-
Other tumors	1	0.47(0.29–0.76)	0.00	-	-	0				
**Gender**										
M only	2	1.16（0.81–1.66）	0.44	0.507	0.0	5	1.08(0.86–1.35)	3.78	0.437	0.0
F only	2	0.73(0.47–1.14)	0.90	0.342	0.0	1	1.09(0.57–2.08)	0.00	-	-
M&F	7	0.55(0.42–0.72)	19.00	0. 004	68.4	5	0.49(0.36–0.66)	7.43	0.115	46.2
**Fractions**										
Tertiles	2	0.76(0.48–1.20)	0.00	0.955	0.0	2	0.78(0.50–1.23)	0.00	0.956	0.0
Quartiles	8	0.59(0.43–0.79)	30.95	0.000	77.4	4	0.50(0.30–0.84)	20.29	0.000	85.2
Quintiles	1	0.92(0.48–1.75)	0.00	-	-	5	1.09(0.84–1.41)	3.77	0.439	0.0
**Number of cases**										
⩽300	6	0.68(0.50–0.91)	4.72	0.452	0.0	4	0.69(0.41–1.17)	6.68	0.083	55.1
301-499	3	0.60(0.29–1.25)	18.28	0.000	89.1	2	0.65(0.24–1.72)	12.81	0.000	92.2
⩾500	2	0.63(0.40–0.98)	9.72	0.002	89.7	5	0.83(0.49–1.43)	26.43	0.000	84.9
**Adjustment for confounders**										
Alcohol										
Yes	2	0.45(0.28–0.71)	1.48	0.224	32.4	6	0.72(0.43–1.19)	30.15	0.000	83.4
No	9	0.70(0.53–0.91)	20.74	0.008	61.4	5	0.76(0.49–1.18)	14.58	0.006	72.6
Smoking										
Yes	6	0.60(0.42–0.85)	29.56	0.000	83.1	8	0.70(0.47–1.04)	43.39	0.000	83.9
No	5	0.66(0.50–0.88)	3.37	0.498	0.0	3	0.87(0.60–1.26)	0.69	0.710	0.0
Body mass index, weight, WHR										
Yes	9	0.61(0.46–0.82)	31.67	0.000	74.7	6	0.58(0.39–0.86)	22.94	0.000	78.2
No	2	0.73(0.47–1.14)	0.90	0.342	0.0	5	1.09(0.84–1.41)	3.77	0.439	0.0
Family history										
Yes	3	0.48(0.40–0.56)	1.34	0.512	0.0	6	0.73(0.46–1.15)	31.00	0.000	83.9
No	8	0.79(0.63–1.00)	10.33	0.171	32.2	5	0.79(0.53–1.18)	8.76	0.068	54.3
Energy intake										
Yes	9	0.73(0.57–0.93)	14.95	0.060	46.5	9	0.91(0.71–1.18)	13.84	0.086	42.2
No	2	0.47(0.37–0.59)	1.34	0.248	25.2	2	0.44(0.37–0.54)	0.49	0.485	0.0

continued

Supplementary Table S2. Stratified analysis of dietary phytosterol consumption and cancer risk.

Supplementary Table S2

Group	Campesterol					Stigmasterol				
	No. of studies	Summary OR(95%CI)	χ^2	*P*	*I*²(%)	No. of studies	Summary OR(95%CI)	χ^2	*P*	*I*²(%)
Total	11	0.72(0.51–1.00)	48.89	0.000	79.5	11	0.83(0.60–1.16)	46.98	0. 000	78.7
**Geographic location**										
Europe	4	1.11(0.70–1.75)	6.71	0.082	55.3	4	1.18(0.69–1.01)	9.44	0.024	68.2
America	6	0.63(0.44–0.90)	12.59	0.028	60.3	6	0.60(0.38–0.95)	18.04	0.003	72.3
Asia	1	0.42(0.34–0.52)	0.00	-	-	1	1.26(1.03–1.54)	0.00	-	-
**Cancer type**										
Digestive system tumors	8	0.72(0.46–1.12)	40.04	0.000	82.5	8	0.92(0.64–1.34)	27.12	0. 000	74.2
Reproductive system tumors	2	0.96(0.68–1.35)	0.34	0.557	0.0	2	0.76(0.26–2.19)	6.25	0.012	84.0
Respiratory system tumors	1	0.47(0.31–0.71)	0.00	-	-	1	0.49(0.32-–0.75)	0.00	-	-
Other tumors	0					0	-	-	-	-
**Gender**										
M only	5	1.09(0.79–1.52)	6.93	0.140	42.3	5	1.21(0.83–1.78)	9.44	0.051	57.6
F only	1	0.81(0.42–1.56)	0.00	-	-	1	0.42(0.20–0.88)	0.00	-	-
M&F	5	0.47(0.37–0.59)	5.47	0.242	26.9	5	0.64(0.36–1.14)	28.66	0. 000	86.0
**Fractions**										
Tertiles	2	0.69(0.44–1.07)	0.15	0.702	0.0	2	0.76(0.48–1.20)	0.16	0.693	0.0
Quartiles	4	0.51(0.32–0.81)	16.95	0.001	82.3	4	0.71(0.38–1.33)	28.32	0.000	89.4
Quintiles	5	1.05(0.71–1.54)	7.61	0.107	47.5	5	0.96(0.54–1.70)	16.76	0.002	76.1
**Number of cases**										
≤300	4	0.59(0.38–0.91)	5.23	0.156	42.6	4	0.51(0.30–0.87)	6.71	0.082	55.3
301-499	2	0.69(0.33–1.48)	6.88	0.009	85.5	2	0.78(0.31–1.96)	9.19	0.002	89.1
≥500	5	0.86(0.45–1.65)	35.73	0.000	88.8	5	1.23(0.88–1.70)	9.44	0.051	57.6
**Adjustment for confounders**										
Alcohol										
Yes	6	0.73(0.40–1.30)	39.31	0.000	87.3	6	0.97(0.61–1.53)	23.78	0.000	79.0
No	5	0.71(0.52–0.97)	7.21	0.125	44.5	5	0.70(0.46–1.07)	11.65	0.020	65.7
Smoking										
Yes	8	0.72(0.47–1.10)	47.72	0.000	85.3	8	0.91(0.61–1.35)	39.14	0.000	82.1
No	3	0.72(0.50–1.05)	0.32	0.854	0.0	3	0.65(0.44–0.95)	1.97	0.373	0.0
Body mass index, weight, WHR										
Yes	6	0.55(0.39–0.79)	19.00	0.002	73.7	6	0.73(0.46–1.17)	29.91	0.000	83.3
No	5	1.05(0.71–1.54)	7.61	0.107	47.5	5	0.96(0.54–1.70)	16.76	0.002	76.1
Family history										
Yes	6	0.77(0.46–1.28)	36.70	0.000	86.4	6	1.02(0.66–1.55)	26.23	0.000	80.9
No	5	0.68(0.45–1.02)	9.19	0.056	56.5	5	0.62(0.36–1.10)	15.15	0.004	73.6
Energy intake										
Yes	9	0.84(0.61–1.15)	20.57	0.008	61.1	9	0.83(0.55–1.24)	30.67	0.000	73.9
No	2	0.43(0.36–0.52)	0.22	0.637	0.0	2	0.80(0.32–2.02)	15.85	0.000	93.7

Supplementary Table S2. Stratified analysis of dietary phytosterol consumption and cancer risk.
